# Supplementary material for: Responsible product design to mitigate excessive gambling: A scoping review and z-curve analysis of replicability
Source: PLoS One. 2021 Apr 20;16(4):e0249926. doi: 10.1371/journal.pone.0249926 (PMC8057587; doi:10.1371/journal.pone.0249926)
Supplement: Attachment — Submitted filename: R23_0322.docx [file pone.0249926.s004.docx]

*Reviewer #2: I appreciate the table but all papers in the table are not as requested in the reference list at the end of the paper.*

**Response: Thank you for raising this point about the revised table and reference list. We have cross-checked the table with the reference list and added any missing papers to the reference list.**

Reviewer #3: In my initial review, I suggested that the authors might consider splitting the paper into two. It seems in their reply that they have indicated that they feel there is not enough on efficacy to be a full paper, and instead their focus is on the replicability and transparency side of things. There seem to be plenty of pages of results about efficacy and maybe three pages about replicability, including a table. In fact, in their reply they say that they are not actually that interested in efficacy per se. The title of the paper has nothing about replicability or transparency, so this isn’t clear. The abstract (which has been rewritten) does seem to cover both sides of things, but I feel that if I went into this study to look for evidence of efficacy, then I’d walk out of it feeling like I’d been deceived into reading about replications instead. Don’t get me wrong, I feel strongly about replication too – it’s crucial to this whole science thing that we’re all trying our best to do. But I’m not sure that I agree with the authors that replication cannot be separated from efficacy for the purposes of a paper, because then you can say that replication cannot be separated from anything and every single paper on anything from now on is going to have to do these analyses. I agree with Reviewer 1, who noted that a lot of this around replication could be an editorial. But I suspect the authors will again rebut this point, and we could go back and forth on this for awhile. I think this will be a call for the editor, and I am of course happy to defer to her views.

**Response: We do appreciate the reviewer's perspective about this issue. However, we remain convinced that keeping the paper as a whole provides a new and interesting perspective about this literature. As we mention in the introduction, other review studies are available, yet these papers do not examine the replicability of this research literature. But we do not wish to mislead readers about the focus of our study, so we have revised our title to “Responsible Product Design to Mitigate Excessive Gambling: A Scoping Review and Z-Curve Analysis of Replicability.” We hope that this revised title better sets readers' expectations and emphasizes our position that replicability is relevant to every discussion of empirical data, and the absence of such discussion might lead readers to make unwarranted assumptions about efficacy. One final note, we would make the same points with regards to the relationship between efficacy and methodological rigor. We did not focus on methodological rigor in the present paper, because (as we noted in the paper) previous reviews have discussed methodological limitations in detail and the state of play has not much changed since then.**

Reviewer #3:The authors have otherwise mostly addressed my concerns, although there are a couple of things that I’ve suggested could be improved throughout, but haven’t been. For example, I pointed out that some studies might have reported BOTH unstandardised and standardised effect sizes, but that other similar things might have been incompletely reported. The authors only addressed the effect sizes example that I highlight. For example, game-based tool or interaction type seems to have some overlap, as percentages add up to more than 100%. There may be other such cases in the paper. As the authors are more familiar with the data, they will be able to identify more of these.

I think the distinction between quasi-experimental and true experimental is actually pretty important, and should be reflected in the new table.

**Response: Thank you for these important points about items reported from each study. We agree with this suggestion and now indicate in Table 2 when a study was quasi-experimental. We also do note in Table 2 when a paper tested more than one type of tool. Also, we would like to kindly remind the reviewer that the first sentence of the “Game-based Tool or Interaction Type” section of the results is “Some studies (n = 7) tested multiple types of responsible product designs.”**

Reviewer #3: I am not sure that I conveyed my concern around the comparison of the Observed and Estimated Discovery Rates. Both of these things have a point estimate, but also a confidence interval around that point estimate. Just because the ODR point

estimate is outside of the EDR confidence interval doesn’t mean statistically significant difference, because the ODR has its own confidence interval and uncertainty which is not taken into account through only using the point estimate of the ODR. The authors, in their revision, have stated that the Observed Discovery Rate is only a point estimate, and that the confidence interval has been omitted, because the sample represents the entire population of interest. I have strong concerns about this, because despite their rigour, there may have been some that were missed for a variety of reasons, or because of errors during search and/or classification, and so on. So, I have a concern with this approach.

To emphasise my point, consider these (obviously made up) data for two groups:

Group 1: 3, 4, 4, 4, 4, 5, 4, 6, 4, 3

Group 2: 6, 3.5, 5, 6, 4, 4, 4, 5, 5, 6

Mean for group 1 is 4.1 (95% CI: 3.47 to 4.73)

Mean for group 2 is 4.85 (95% CI: 4.17 to 5.53)

So the estimate for each is not within the CI of the other.

If I forget that group 2 has a CI, and just compare 4.85 to the CI for group 1, I would conclude that there is a statistically significant difference.

But there is a CI for group 2, and if I run an independent samples t-test (Welch or standard), the p-value is .082, so we would conclude no significant difference. Admittedly, it’s close, but if we’re to use an alpha criterion of .05 for p-values (as absolutely no one ever intended them to be used, and that’s a discussion for another day), it’s not the same as just comparing a single point estimate against a confidence interval, which is what is done in the paper. Given that this is used as evidence for a talking point by the authors, this is pretty important. If this is how the author of the z-curve suggests that the tool is used, then I have hesitations about the procedure, and this harks back to my concerns from my initial review about using new techniques.

**Response: We now note in the limitations section that accidentally omitting relevant studies “might have affected the conclusions we drew about replicability and publication bias based on the results from z-curve.” That said, after reflection on your comment, we do not think that omitting the confidence interval of the Observed Discovery Rate is one of the potential weaknesses of z-curve. Although it is true that we might have overlooked some hard-to-find studies, we do not**

**believe that the confidence interval would adequately represent this source of uncertainty. Rather, the confidence interval represents uncertainty from having analyzed a random sample from a larger population. For instance, reporting the confidence interval around the Observed Discovery Rate would have been appropriate had we decided that coding the *p*-values from all studies was too much work, and so we deliberately only coded a random subsample of them. The source of uncertainty presently in question is not random—there would have to be something about the study(ies) that made it (them) hard to find or difficult to correctly classify as relevant, which might in turn be related to its (their) replicability.**
